# Rapid Sampling of Tomato Juice Using a 3D-Printed Microneedle Assisted by an Unmanned Aerial Vehicle

**DOI:** 10.3390/mi17101184

**Published:** 2026-10-13

**Authors:** Weixin Liu, Tianyu Wang, Jiahua Zhong, Qinghao He, Haonan Li, Weijin Guo

**Affiliations:** 1 Department of Electronic Engineering, Shantou University, 243 Daxue Road, Shantou 515063, China; 2 Department of Biomedical Engineering, Shantou University, 243 Daxue Road, Shantou 515063, China; 3 School of Biomedical Engineering, Shenzhen Campus of Sun Yat-Sen University, 66 Gongchang Road, Shenzhen 518107, China

**Keywords:** UAV, UWB, microneedle, 3D printing, synthetic paper, capillary sampling, maturity monitoring

## Abstract

Extracting tomato juice for maturity assessment is critical for ensuring a timely harvest. Nevertheless, the limitations of existing sampling tools and heavy reliance on manual operation may result in tomatoes being harvested outside their optimal window, resulting in economic losses. Therefore, there is an urgent need for a better method for tomato juice sampling and maturity monitoring. In this work, an unmanned aerial vehicle (UAV)-mediated directional sampling system (uDSS) was developed for tomato identification in the field and the collection of tomato juice. The system integrates a UAV with a real-time ultra-wideband (UWB) positioning system. The system autonomously performs the flight and juice-sampling sequence; juice recovery and Brix measurement are carried out after the flight. With regard to the sampling procedure, a 3D-printed microneedle (MN) with porous synthetic paper for capillary liquid absorption was employed to penetrate the tomato and extract juice. Lastly, the sugar content of tomatoes was measured in order to verify the feasibility of uDSS. This unmanned system is expected to promote the development of UAV-based solutions in precision agriculture and intelligent food industry.

## 1. Introduction

Crop production is of fundamental importance to human survival and development [1,2]. The growth status and quality of crops are closely related to food security and yield. The ability to accurately identify the composition of crops is of paramount importance for discovering any unfavorable factors at an early stage, such as changes in maturity, diseases and pests, or environmental pollution [3,4]. This information can then be used to guide timely harvesting or implement targeted prevention and control measures, in order to ensure crop yield and quality, and to meet the growing demand for food globally.

In general, conventional agricultural management employs a unified fertilization or manual harvesting approach, contingent on macrolevel factors such as climate, topography and soil, which are susceptible to natural resource wastage and diminished profit efficiency [5]. There are two kinds of traditional methods for monitoring the maturity of crops. The first method is manual observation, which depends on subjective judgement based on appearance characteristics or tactile sensations. The second method involves the utilization of tools, such as cutting of the stem or fruit, to facilitate the observation of the internal structure. Despite subjective judgement and tool accessibility, these methods also require substantial time and manual effort investment [6].

Recently, with the development of intelligent systems and manufacturing technology, unmanned aerial vehicles (UAVs) have become a powerful tool in many fields, including military reconnaissance [7], urban environmental monitoring [8], agricultural production [9,10,11] and aerial photography [12], whilst the scope of application is being expanded [13,14]. Since the beginning of the 21st century, the popularity and development of UAV technology in the field of agriculture have accelerated [15], with many agricultural UAVs emerging, primarily for the purposes of pesticide spraying [16], sowing and fertilization [17]. Primicerio et al. proposed the six-axis aerial platform UAV for vineyard management and development [18]. Göktoǧan et al. developed the agricultural UAV, designated ‘RUAV’, for the management and detection of aquatic vegetation [19]. Zhang et al. invented a methodology for predicting the yield of soybean crops, which involved the use of a hyperspectral DJI Spreading Wings S1000+ UAV [20]. Fahmi et al. used the UAV ‘X-Copter’ for the detection of palm oil plantations [21]. An intelligent agricultural UAV integrating Internet of Things (IoT) technology was developed to facilitate the process of crop spraying [22]. Spoorthi et al. proved that the ‘Freyr UAV’ could be controlled by an Android application, thereby facilitating the spraying of pesticides and fertilizers [23]. However, the application of UAVs in crop maturity monitoring remains limited [24], and most reported systems are restricted to non-contact optical sensing of the canopy, which provides only indirect and largely surface-level information about fruit maturity. Furthermore, automated sampling of fallen fruit—a critical indicator for crop health and early pest/disease warning—remains an unresolved challenge. Consequently, there is an urgent need for a more advanced automated and intelligent sampling method that can physically access crop fruit and collect its juice directly, including for fruits that have fallen to the ground.

Tomato is an important economic crop that is extensively cultivated across the earth. It exhibits remarkable environmental adaptability. In the context of tomato cultivation, the sugar content is a pivotal metric for assessing its degree of maturity [25]. As the tomato matures, its internal starch undergoes a gradual transformation into soluble sugar. Generally, an increase in soluble sugar content is associated with a more favorable taste and increased economic value. Consequently, precise and rapid determination of tomato sugar content is critical for the determination of optimal harvest time, the assurance of tomato quality, and the maximization of economic benefits. Maturity measurement based on juice composition analysis is a core method for fruits and vegetables, including tomatoes. Its fundamental procedure is the extraction of juice samples by physical means, such as puncture and extrusion. After that, a quantitative analysis is conducted on the concentration of specific elements or compounds (such as soluble sugar), enabling the determination of tomato maturity. Current methods employed in juice analysis for evaluation of maturity or quality include Brix value measurement, sugar concentration measurement, pH value detection, and near-infrared spectrum detection, amongst others [26,27,28]. However, the majority of these methodologies still rely on the manual collection of juice samples for subsequent analysis [29]. Image-based color measurement has also been used to characterize the appearance of agricultural foods [30].

As an emerging microtechnology, microneedles (MNs) have been developed for minimally invasive liquid collection from biological surfaces [31,32,33]. In biomedical engineering, MNs allow interstitial fluid or blood to be accessed with minimal pain and trauma, and MN-based sampling has recently been extended to plants and crop health monitoring [34]. Compared with conventional puncture-and-extrusion tools, a microneedle provides a well-defined penetration depth and a closed liquid path, so that a small and reproducible volume of fluid can be withdrawn directly from the target tissue with limited damage to the fruit. For plant fluids, which are more viscous and contain solid particulates, a relatively large tip diameter and an open capillary conduit are required to avoid clogging, and the mechanical properties of the needle must be matched to the toughness of the fruit skin [32]. Fabrication of such needles has been demonstrated by micromolding, lithography and laser micromachining [33]; however, these cleanroom-based routes involve multiple processing steps, dedicated masks or molds, and long design-to-device cycles, which makes it difficult to rapidly iterate the needle geometry for a new target fruit. Additive manufacturing removes most of these constraints, because a custom geometry can be printed directly from a digital model at low cost and in a short time [35]. All-inkjet-printed conductive microneedles have also been demonstrated for monitoring plant electrochemical properties [36]. In this work, the microneedle is, therefore, regarded as a microfabricated, application-specific sampling interface rather than a simple puncture tool, and its geometry is designed to match the mechanics of tomato skin and to meet the capillary demands of synthetic paper. Sheng et al. developed a microneedle-based emergency drug delivery system assisted by a UAV and verified its effectiveness on pigs in the jungle [37]. In that system, the microneedle acts as a delivery interface and is used in a single, manually supervised operation. In contrast, the present work uses the microneedle as a sampling interface: the needle must penetrate a small, mobile and visually localized target, collect a measurable volume of juice through capillary action, and be retracted by the UAV without loss of the sample. A comparison of the proposed uDSS with representative UAV-based systems for agriculture and aerial interaction tasks is summarized in Table 1.

Taken together, the contribution of this work is an integrated unmanned sampling system rather than any single component. The individual building blocks, namely UWB positioning, visual inertial odometry, trajectory planning and YOLOv5 detection, are mature technologies, and the novelty pursued here lies in the integration of a 3D-printed microneedle sampling interface with an autonomous aerial platform: the microneedle must be actuated by the vehicle itself, its penetration event must be detected in real time from a thin-film pressure sensor and converted into a control decision, and the collected juice must be retained by the synthetic paper during the return flight. This work, therefore, addresses the mechanical and control problem of operating a microfabricated sampling interface from a flying platform, whereas previous UAV-based agricultural systems have been used mainly for non-contact sensing or for the release of agrochemicals. The microneedle, its fabrication route and its capillary coupling to the synthetic paper are consequently central to this study, and the UAV is the platform that demonstrates their use in the field; accordingly, the individual design choices of the needle are reported explicitly in Section 2.3. It should be noted that the needle used here is not a micron-scale microneedle in the usual sense: the tip diameter of 0.2 mm and the width of the paper slots are micro-scale features, whereas the total length of 30 mm is dictated by the mechanical interface of the vehicle rather than by the biology of the fruit. The term microneedle is retained here because the needle provides a minimally invasive, capillary-based sampling interface of the type that is normally realized with microneedle technology and because the printed tip is the functional micro-feature of the device. These dimensions are, therefore, stated explicitly in Section 2.3, together with the design rationale for each, so that there is no ambiguity about the scale of the device.

**Figure 1:**
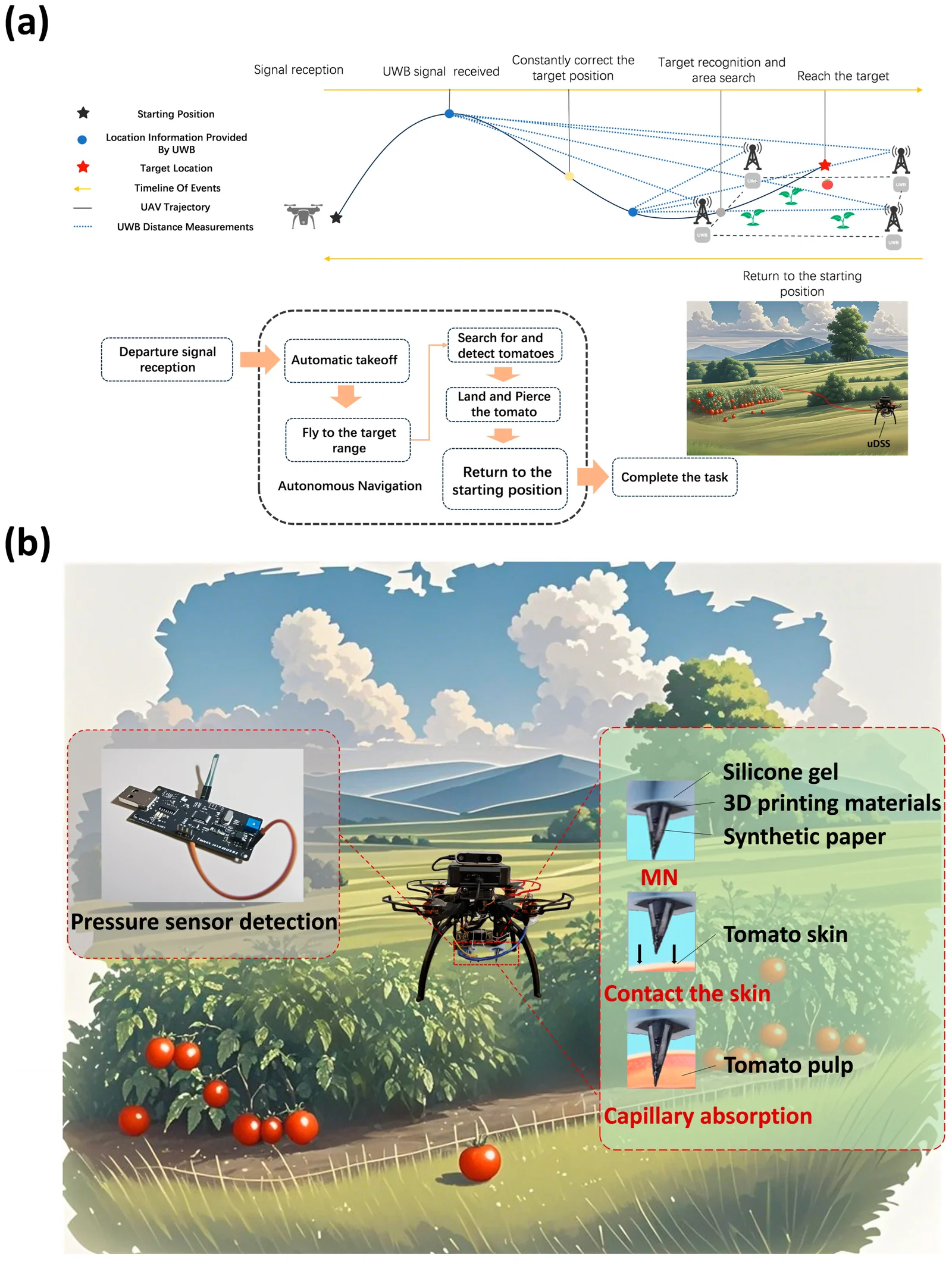
uDSS workflow and MN device diagram. (**a**) Overall workflow of the uDSS. Upon receiving the start signal, the UAV takes off, navigates to the task field using UWB positioning, identifies the target tomato with the on-board detection algorithm, descends and perforates the fruit with the MN device, collects the juice, and returns autonomously to the starting position; the four numbered stages correspond to (1) take-off and navigation, (2) target recognition, (3) descent and juice sampling, and (4) return. (**b**) Working principle of the MN device: the tapered 3D-printed microneedle first contacts and penetrates the tomato skin, and the tomato juice is then drawn into the hydrophilic porous synthetic paper through the paper slots on the needle shaft by capillary action.

The detailed UAV sampling process is illustrated in Figure 1. Initially, a testing field was set up outdoors and tomatoes were placed in various locations within this field to simulate the tomato field. Subsequently, the sampling signal from the tomato field was transmitted to the UAV via the UWB real-time positioning module. Then, the UAV flew to the target area based on the environmental conditions, employing the YOLOv5s detection algorithm to precisely identify and localize the target tomato. Next, the UAV descended, and the MN device affixed beneath it penetrated into the target tomato. The tomato juice was then sampled using synthetic paper (with high water absorption capacity) connecting the MN device and was also stored in synthetic paper. After sampling was completed, the UAV was programmed to transport the sample to a designated location. The synthetic paper was separated from the MN device and then subjected to centrifugation in a centrifuge tube, thus yielding the collected juice samples. The Brix value was measured using a sugar meter to determine the tomato maturity and verify the performance of uDSS. Owing to its high mobility and environmental adaptability, the UAV was successfully used to monitor the maturity of the sampled tomatoes. We believe this work can provide some guidelines for unmanned crop maturity monitoring in the near future.

## 2. Experiments and Results

### 2.1. Materials and Equipment

The printed circuit board (PCB) of uDSS is designed and drawn by the Jialichuang EDA software (v2.2.37.3, Shenzhen, China). After the PCB design is completed, it is produced by Jialichuang (Shenzhen, China). Pressure sensor is from Wuxi Aiding Electronics (RP-C10-ST, Wuxi, China). UAV frame is from Volcano Crater Model (Q250, Dongguan, China). UAV flight control system is provided by Heli Brothers (Holybro Pixhawk 6C, Shenzhen, China). UAV motors are from Huaxintong Trading (HKSRC 2205-2450KV, Shenzhen, China). Electric motor for the UAV is from Dongfang Haoyue (Formula 32-bit 45A ESC, Shenzhen, China). Onboard computer is from Intel (Intel BXNUC10i7FNKN2, Shanghai, China). Depth camera is from Intel (Intel RealSense D435i, Santa Clara, CA, USA). UWB module is from NoopLoop (LinkTrack S, Shenzhen, China). USB camera is from Heda (DSJ-YC60Z, Shenzhen, China). Distance measurement sensor is from Wheeltec (STP-23, Dongguan, China). 3D printer is from Creality (K1, Shenzhen, China). Brix detector is from DiFluid (R2 PP, Shenzhen, China). The detailed costs of components and consumables used in uDSS are listed in Table S1.

### 2.2. Construction of UWB Real-Time Positioning System

The UWB real-time positioning system uses ultra-wideband technology for accurate position tracking. The transmission of the signal is facilitated by high-frequency radio waves, and the precise location of the object is obtained by the calculation of propagation time difference of the received signal [38].

**Figure 2:**
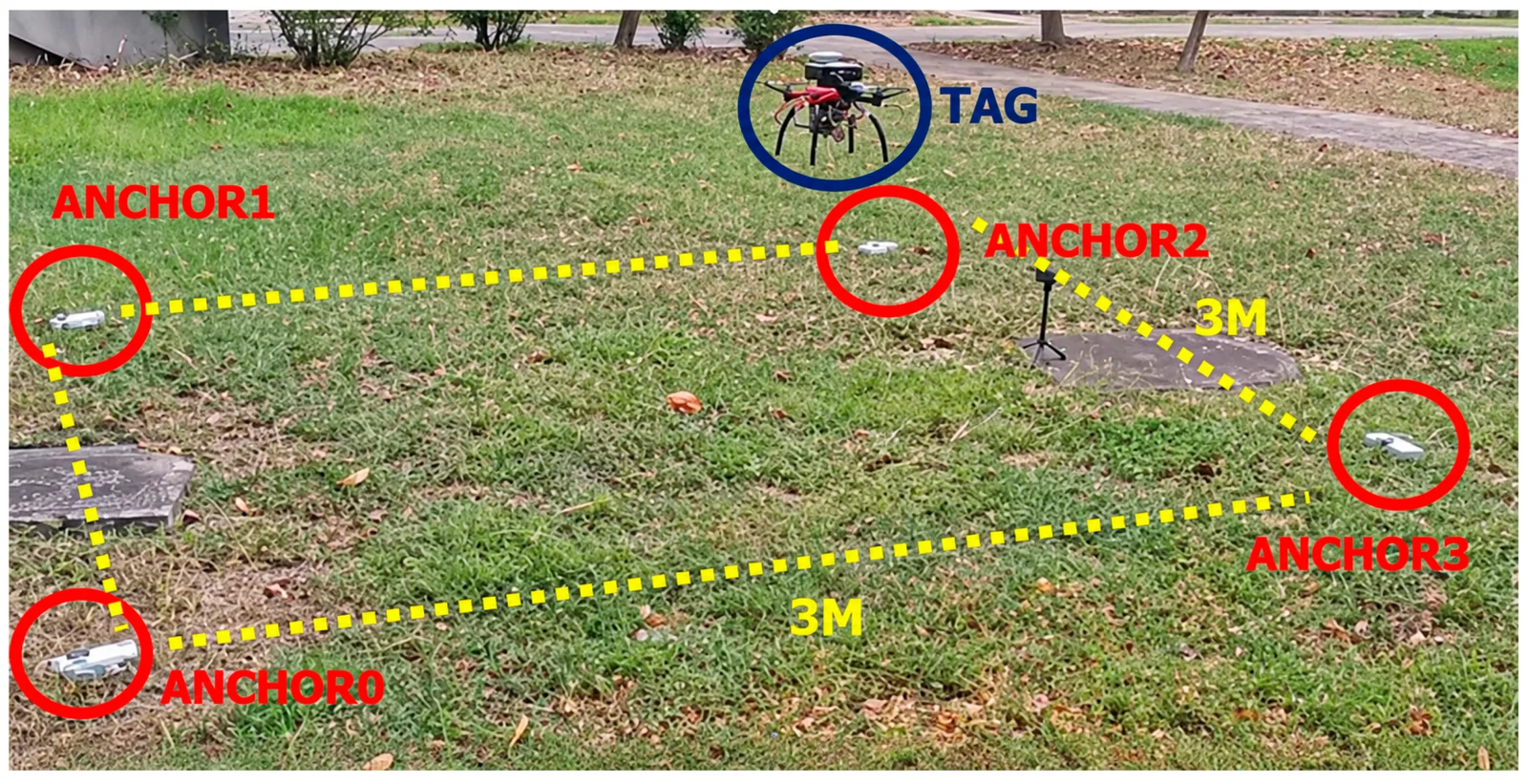
Layout of TAG and ANCHOR in the experiment.

A 3 m × 3 m outdoor area was chosen for the proof-of-concept experiments, based on the available space and a four-corner UWB anchor layout. The area accommodated separate tomato targets to simulate the distribution of fallen fruit and allowed the navigation, target recognition and juice-sampling sequence to be demonstrated under controlled conditions. Thirty static positioning tests at known coordinates within this area yielded a mean positioning error of 10.8 ± 5.8 cm (mean ± standard deviation). This result characterizes the positioning performance of the tested anchor arrangement; operation over larger areas would require further evaluation. The configuration of UWB real-time positioning system within the tomato field is illustrated in Figure 2. The ANCHOR component is installed at a predefined fixed point, designated as the reference position. The TAG component is installed on the UAV, serving as the node to be positioned. The CONSOLE component (connected to the computer) is deployed to oversee the operational status of the system and to transmit commands to the ANCHOR, TAG, and other nodes, thereby facilitating communication with the terminal equipment. After obtaining the real-time position in the constructed spatial coordinate system, the UAV can use the target point given by the motion control module of the UAV to realize the navigation function and thereafter the coverage search function in the task area. In this work, the ANCHOR is arranged at the 4 corners of the rectangular tomato field. When the UAV comes close to the area surrounded by the ANCHOR, the positioning source of the UAV is switched to the real-time positioning information obtained by TAG to ensure that the movement of the UAV can be accurately controlled during the tomato recognition.

### 2.3. Manufacturing of uDSS

**Figure 3:**
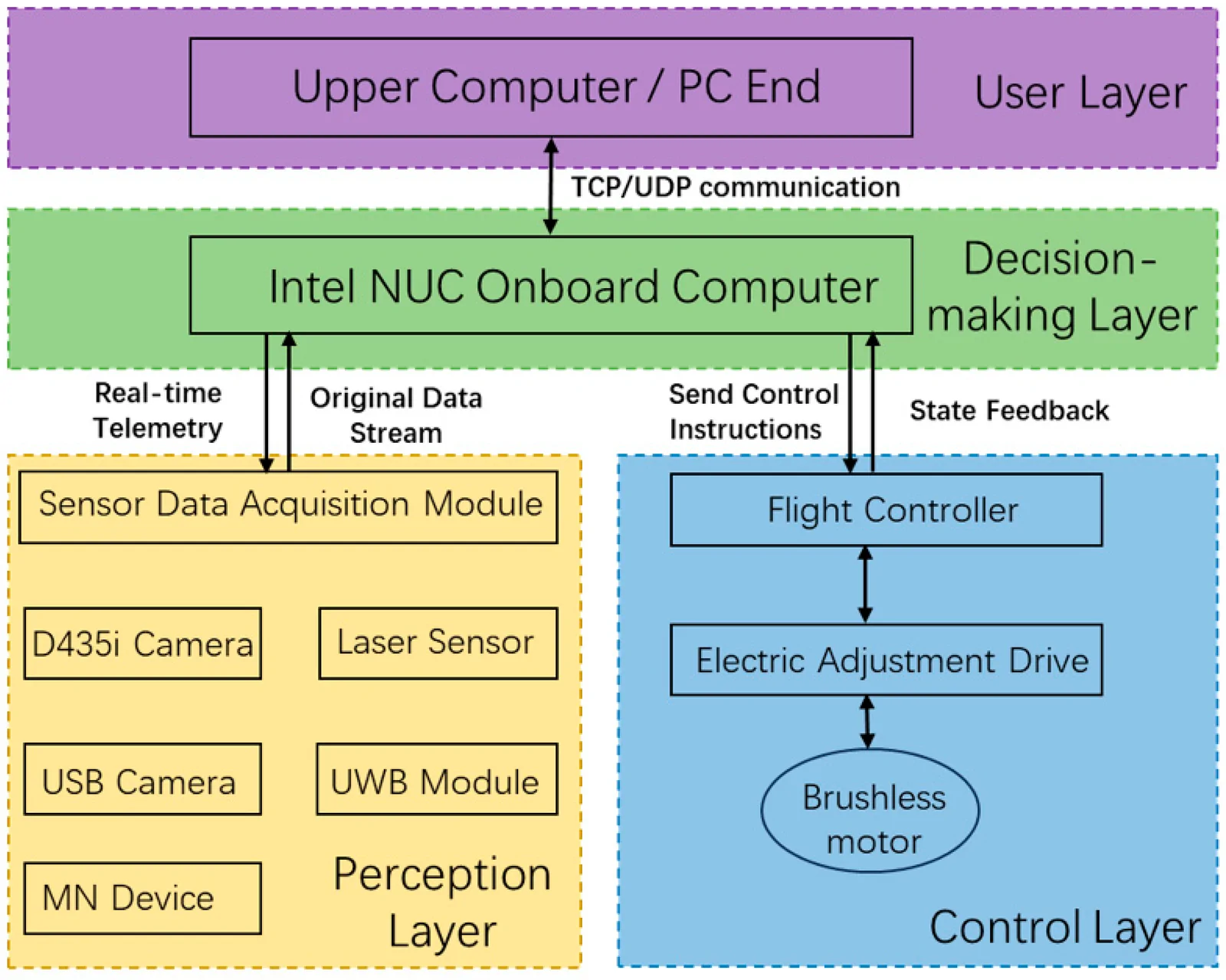
The overall architecture of uDSS, including user layer, decision-making layer, perception layer, and control layer.

The operation logic of uDSS is based on a four-layer architecture, comprising the user layer, decision-making layer, perception layer, and control layer, with the system structure illustrated in Figure 3. During actual operation, the user layer is responsible for monitoring the system’s status and issuing start and stop commands. This layer establishes a remote connection with the onboard computer in the decision layer. The onboard computer receives multi-source data from the perception layer, including camera images, pressure sensor signals, and UWB positioning information, and processes these data through predefined algorithms for fusion and analysis. It then generates control commands and sends them to the flight controller in the control layer. The flight controller adjusts the drone’s flight attitude and trajectory in real time based on the instructions to precisely complete the sampling task in the target area. Meanwhile, the pressure sensor continuously monitors pressure change on the MN device and feeds the data back to the onboard computer, thereby forming a closed-loop autonomous control system. This architecture not only significantly enhances the drone’s autonomy and operational flexibility but also improves its adaptability and robustness in complex environments.

#### 2.3.1. The Composition and Construction of UAV

**Figure 4:**
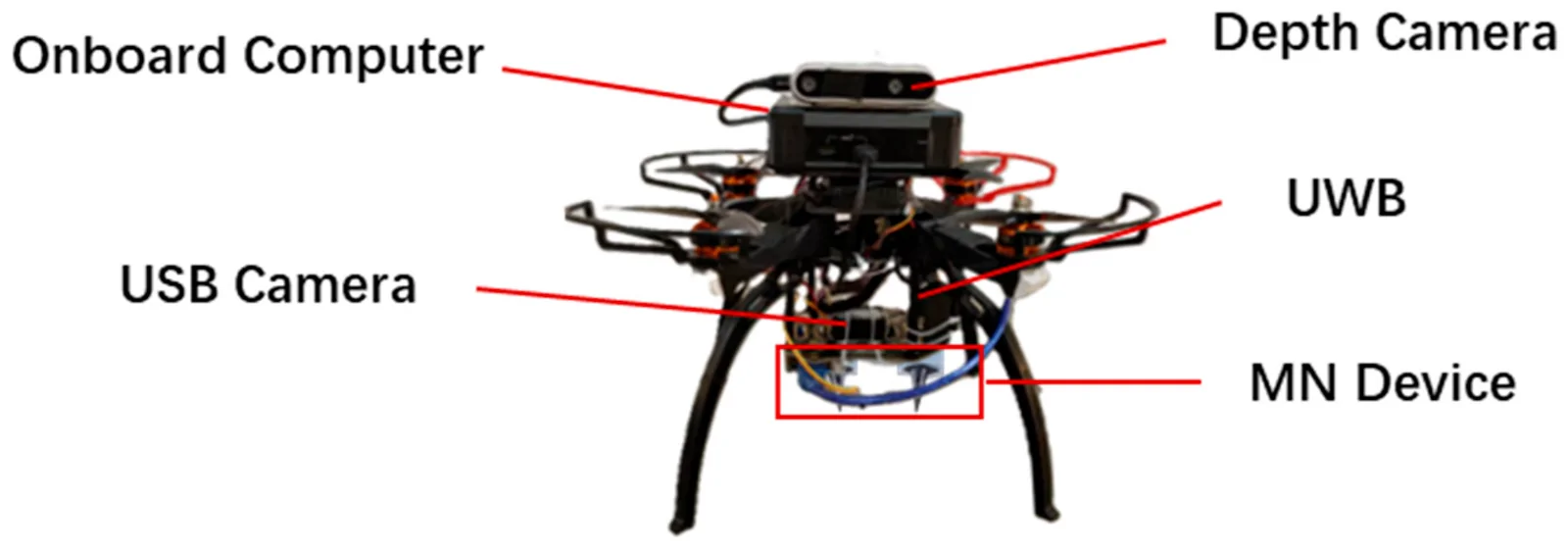
Four-axis UAV in uDSS, which mainly consists of an onboard computer, a depth camera, UWB, MN devices, and a USB camera.

In this work, we used a four-axis UAV platform that was independently designed and constructed. The platform is systematically designed based on actual task requirements and features a high degree of customizability and flexible operation. The UAV’s autonomous control system consists of five core modules: an upper-level onboard computer, a lower-level flight control terminal, an environmental information sensing unit, a positioning information module, and an MN device. The overall architecture is shown in Figure 4.

In this system, the onboard computer is responsible for high-intensity computing and task decision making; the flight control terminal focuses on the attitude stabilization of the UAV itself; the environmental perception unit is composed of a depth camera and a USB camera, which are used for depth visual perception and target search within the field of view, respectively; and the positioning information module relies on UWB sensors to obtain real-time precise position information of the target tomato field. The flight controller, sampling devices, and other sensors are all connected to the onboard computer, enabling real-time data processing to ensure high task efficiency.

#### 2.3.2. MN Device Fabrication

**Figure 5:**
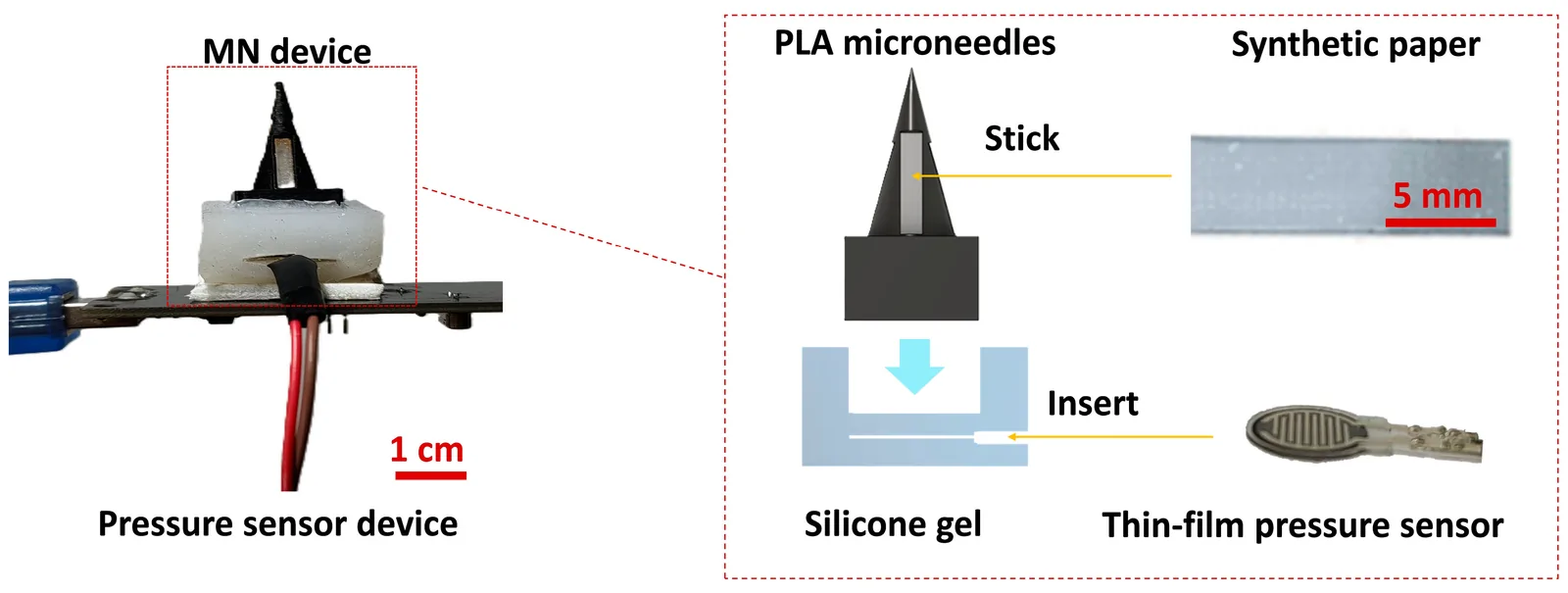
An MN device and its components: a pressure sensor, synthetic paper, silicone gel base, and a 3D-printed microneedle.

The MN device adopts a composite system that can detect the pressure of tomatoes on microneedles in real time and collect and store tomato juice. It mainly includes 3D-printed microneedles, synthetic paper, a silicone base and pressure sensor system. Its design and components are shown in Figure 5. First, the microneedle was 3D modeled using Fusion 360 software (Autodesk, San Francisco, CA, USA) and printed with a commercially available fused-deposition-modelling (FDM) 3D printer (Creality K1) using polylactic acid (PLA) filament. The printing parameters were a nozzle diameter of 0.4 mm, a layer height of 0.1 mm, an extrusion width of 0.42 mm, an extrusion temperature of 210 °C, a bed temperature of 60 °C and a printing speed of 60 mm/s; the needle was printed vertically so that the layer boundaries are perpendicular to the penetration direction. FDM was selected in preference to micromolding, lithography or laser micromachining because it allows the needle geometry to be modified directly from the CAD model, so that the tip diameter, the taper angle and the position and size of the two synthetic paper slots could be iterated between sampling trials at negligible cost and within a few hours, which is essential for a proof-of-concept study in which the sampling interface must be adapted to the target fruit. The printing parameters define the fabrication conditions used for this prototype. To balance low insertion resistance and ensure thorough contact between the synthetic paper and the juice, a tapered needle tip configuration was adopted; the taper also distributes the puncture load over a longer section of the needle, which reduces the risk of buckling at the tip. The CAD design specifies a total length of 30 mm, a needle length of 19.72 mm and a tip diameter of 0.2 mm. These dimensions follow from the sampling requirements rather than from the limits of the printer. The overall length of 30 mm is set by the distance between the mounting flange at the bottom of the UAV and the lowest point of the vehicle, so that the needle can reach the fruit while the propellers and the landing gear remain clear of the tomato and of the ground. The needle length of 19.72 mm is the maximum length that leaves the two synthetic paper slots fully exposed above the puncture point while keeping the needle slender enough to be inserted by the available thrust. The nominal tip diameter of 0.2 mm was selected to provide a tapered contact region for puncture. These dimensions are CAD design values. The printer settings describe the fabrication process and do not establish the measured dimensions or dimensional repeatability of the printed parts. Functional sampling performance is reported in Tables S2 and S3; quantitative dimensional and mechanical characterization remains a subject for further work.

Two synthetic paper slots are integrated into the needle shaft sides, with design dimensions of 10.79 mm × 2.5 mm and 12.23 mm × 2.5 mm, respectively. The two slots are deliberately arranged at different heights and on opposite faces of the shaft. The two nominal slot areas sum to 57.55 mm^2^. Applying the measured areal absorption capacity of 18.24 ± 1.67 µL/cm^2^ gives an estimated capacity of approximately 10.5 µL for an equivalent exposed paper area; this estimate does not determine whether the paper reaches saturation during sampling. Synthetic paper is a hydrophilic porous medium with interlocked micropillars made by OSTE multi-directional lithography, and the preparation materials and methods are based on previous work [39]. We conducted fundamental performance tests on synthetic paper. The absorption performance of the synthetic paper was evaluated as follows (the measurement procedures are described in detail in the Supplementary Information, Section “Performance measurement of the synthetic paper”). A 1.0 cm^2^ synthetic paper sample was brought into contact with a reservoir of freshly extracted tomato juice; the filling rate was determined from the increase in mass of the sample recorded with a balance (0.1 mg resolution) at 1 s intervals, and the filling rate is defined as the ratio of the volume of juice absorbed within 5 s to the total pore volume of the sample. The same experiment gave an average tomato juice absorption capacity of 18.24 ± 1.67 µL/cm^2^. After centrifugation at 3000 rpm for 5 min, the average tomato juice recovery rate, defined as the ratio of the volume of juice released by centrifugation to the volume of juice previously absorbed by the sample, was 96.15 ± 0.53%, ensuring sufficient sample volume for subsequent testing and analysis. The pore size and the interlocked micropillar geometry of the synthetic paper are determined by the OSTE multi-directional lithography process and were characterized in a previous work [39]; the interconnected structure supports capillary uptake. Tomato juice absorption and recovery were measured directly under the conditions described in the Supplementary Information. Its 3D structural diagram is shown in Figure S1. Its porous structure can effectively absorb and temporarily store tomato juice via capillary action. After the tomato juice is absorbed by synthetic paper, the juice can be collected by centrifugation for following analysis in a lab. For the thin-film pressure sensor system, the overall architecture is illustrated in Figure S2, which consists of three functional modules responsible for data transmission, data processing, and data acquisition. In addition, the power supply and serial signal conversion module ensures stable power supply and enables data communication with the onboard computer, with its circuit schematic shown in Figure S3. The CH340C circuit for serial communication is shown in Figure S4. The physical design of the pressure sensor is shown in Figure S5. To evaluate the influence of sensor dimensions on measurement performance, puncture experiments were conducted on the same tomato using sensors of different diameters, and the experimental data were analyzed, as shown in Figure S6. Furthermore, a voltage comparator module was integrated to enable threshold-based pressure detection, where the experimental process could be visually monitored through the on/off state of an LED indicator. The corresponding circuit schematic is shown in Figure S7. We evaluated sampling and puncture outcomes for tomatoes of different sizes and ripeness levels, as summarized in Tables S2 and S3, which provide a descriptive assessment based on a limited number of fruits with repeated trials. Subsequently, the prepared synthetic paper was pasted on the surface of the 3D-printed microneedle and placed in a silica gel base. Lastly, a thin-film pressure sensor was inserted into the silica gel base to complete the assembly of the MN device.

### 2.4. Sampling of Tomato Juice

For testing, a tomato was placed within the 3 m × 3 m outdoor test area. The UWB base station (ANCHOR as shown in Figure 2) was then deployed at four corners of the field, with the purpose of providing the target location information for the uDSS. The UAV is located at the starting position, situated 10.0 m from the tomato. The open-source visual inertial odometry (VIO) framework is utilized to facilitate the self-positioning of the UAV [40], and the trajectory control of uDSS is based on the enhanced and optimized fastplanner algorithm, which facilitates the expeditious and effective realization of the target field position [41]. The planner employs kinematic path search to find an initial collision-free path, then optimizes the path using B-spline optimization combined with gradient information from the Euclidean distance field, thereby pushing the trajectory away from obstacles. Finally, iterative time adjustment ensures the dynamic feasibility of the trajectory. This three-layer architecture enables the planner to generate safe, smooth, and feasible trajectories in complex environments with extremely short computation times (typically within 100 ms). The planner provides the trajectory generation component of the sampling system; physical and dynamic obstacle avoidance was not evaluated in the field trials in this work.

Upon reception of the sampling signal, the UAV equipped with the MN device is programmed to take-off immediately. Based on the target field position signal and the obstacle map constructed from the real world, the automatic trajectory planning with obstacle avoidance capability can be realized. Subsequently, through autonomous navigation, the UAV will proceed to the designated tomato field. Concurrently, the signal source is continuously repositioned through the onboard UWB sensor to obtain accurate positioning information. YOLOv5s was used for tomato identification and localization on the onboard Intel NUC computer [42]. The detected fruit coordinates were supplied to the flight control module to align the UAV above the target tomato before descent and sampling. The tomato image dataset comprised 894 annotated images with a single object class, tomato. The detection evaluation yielded an mAP@0.5 of 97.1%, a precision of 93.5%, and a recall of 91.7%. These point estimates describe the evaluated configuration. The altitude information is provided by the laser rangefinder module, and the PID control algorithm is then applied to facilitate a slow and smooth landing.

During the drone descent and sampling phase, the system employs a logical architecture integrating hierarchical precision control with autonomous decision making. At the control level, the horizontal direction uses the deviation between the target’s pixel coordinates in the camera frame and the image center as input, while the vertical direction utilizes the real-time altitude feedback from the laser ranging module as control input. The PID coefficients employed are as follows: proportional gain Kp = 1.0, derivative gain Kd = 1.78, integral gain Ki = 0.005. The control strategy adapts based on altitude: in the high-altitude phase (>0.8 m), rapid coarse adjustment using position PID control with 0.5 m horizontal steps; during the mid- to low-altitude phase (0.3–0.8 m), the horizontal step size is reduced to 0.05 m, with a stepwise vertical descent at 0.15 m intervals; in the precision ground phase (≤0.3 m), the control step size is further refined to 0.02 m for final alignment. Subsequently, the system switches to velocity control mode, rapidly descending to the ground at 0.4 m/s to complete the penetration. Simultaneously, the onboard computer continuously monitors pressure sensors.

Based on prior independent penetration tests, the force threshold used in the sampling control sequence was set at 1.5 N for the tomatoes tested in this work. The pressure sensor signal was monitored by the onboard controller, and an adjustable-voltage comparator provided the trigger for the subsequent juice-absorption step. Figure S6 compares the responses of sensors with three different diameters. The threshold would require further evaluation before application to other tomato cultivars or ripeness ranges. If the signal exceeds this threshold, penetration is deemed successful, triggering a 30 s juice absorption process before automatic return. Otherwise, the UAV automatically ascends and retries. The complete sampling missions achieved a 100% success rate under the tested conditions, with automatic retries permitted. This mission-level result is distinct from first-attempt sampling success in Tables S2 and S3. It should be noted that the collision-free constraint is embedded in the trajectory planner, which evaluates the Euclidean signed distance field of the environment; the field trials reported here were carried out in an open outdoor field, in which no physical obstacle interrupted the flight path, so the avoidance behavior was verified at the level of the planner rather than against a physical obstacle, and the response to dynamic obstacles was not tested. An experimental evaluation of avoidance against physical and dynamic obstacles will be pursued in future work.

**Figure 6:**
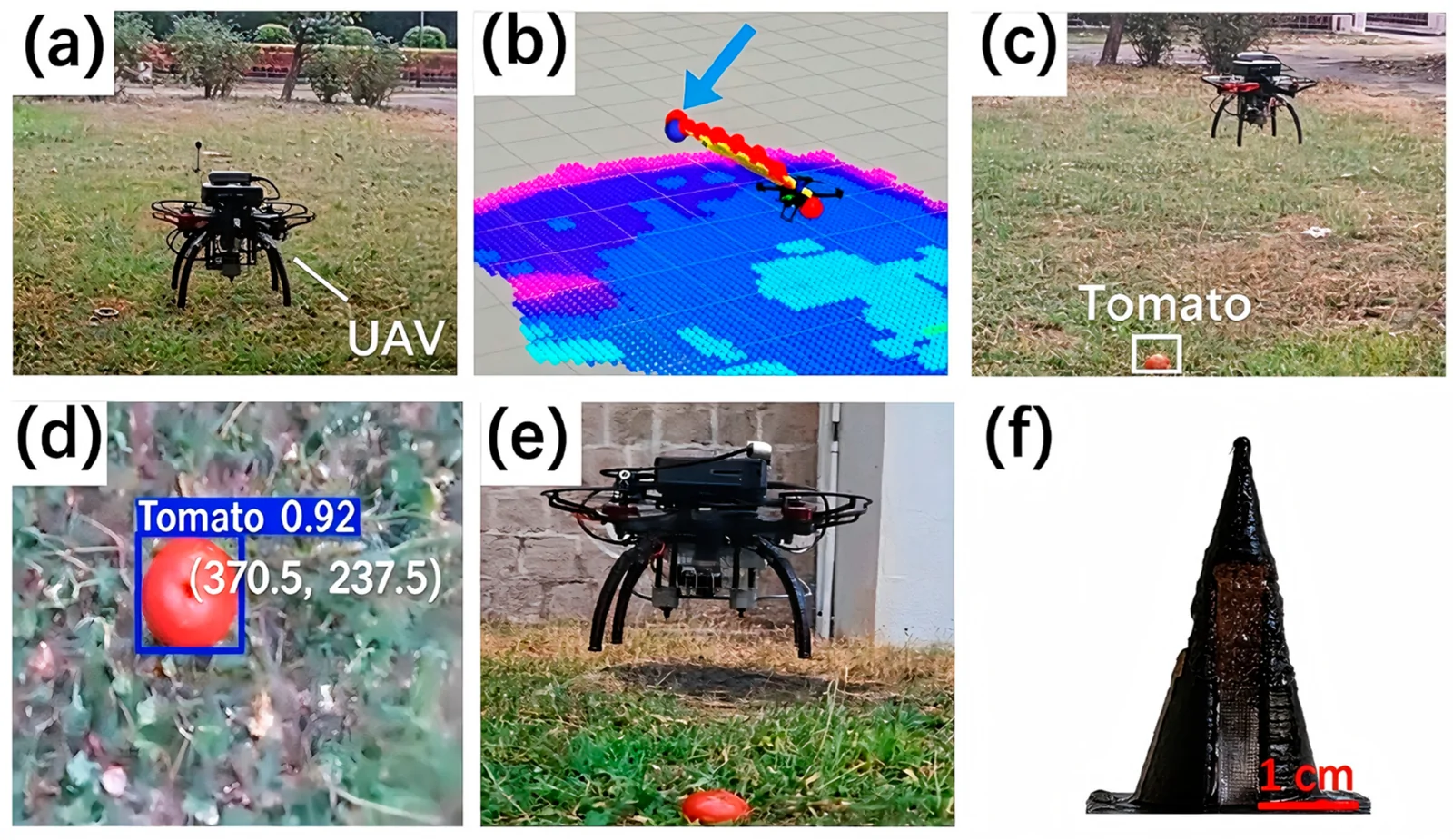
The sampling of tomato juice. (**a**) When the sampling signal is received, the UAV takes off autonomously. (**b**) The UAV goes to the target point by automatic trajectory optimization. The red line is the optimized trajectory, and the blue point is the target point obtained after UWB calculation. (**c**) The UAV reaches the target field. (**d**) Target recognition is performed. (**e**) After the UAV recognizes the target, it starts descending for sampling. (**f**) Tomato juice is collected by the MN device and stored in synthetic paper.

Following the implementation of puncture sampling, the synthetic paper was kept in contact with the tomato juice for 30 s. Ultimately, the UAV autonomously takes off and returns to the starting position. The operational efficiency of uDSS was assessed by timing 10 complete sampling cycles. The average total time from take-off to return after sample collection was 2.5 ± 0.3 min. This duration includes autonomous navigation to the target, tomato recognition, precision descent, 30 s juice absorption, and return to base. The detailed process is illustrated in Figure 6. Tables S2 and S3 summarize the sampling and puncture outcomes. The subsequent Brix measurements demonstrate that juice recovered after aerial sampling can be used for an offline compositional measurement.

### 2.5. Measurement of Tomato Sugar Content

**Figure 7:**
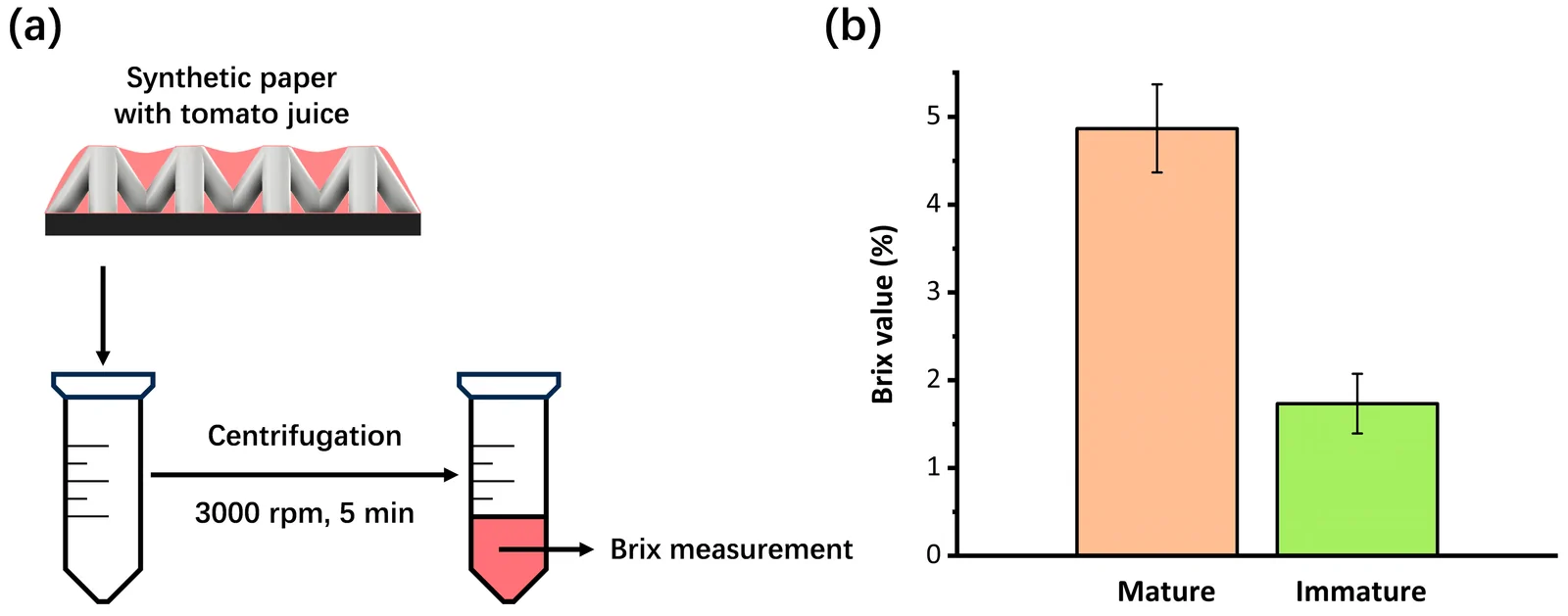
Tomato juice recovery by centrifugation (**a**) and sugar content measurement by a Brix detector (**b**).

In order to verify the effectiveness of uDSS for tomato juice sampling, the MN device containing the juice was removed after sampling, and the synthetic paper was separated to recover the tomato juice. The synthetic paper was placed in a centrifuge tube and centrifuged at 3000 rpm for 5 min to obtain tomato juice [39], as shown in Figure 7. A fresh piece of synthetic paper was used for each sampling, to reduce the risk of carryover between samples. The Brix value can be used to refer to sugar content and has been validated to be related to tomato maturity [43]. A Brix detector was used to measure soluble solids in the recovered juice. Two tomatoes at different maturity stages were tested as a demonstration. The comparison illustrates the feasibility of recovering juice for offline Brix measurement; it does not systematically establish the accuracy of maturity classification.

## 3. Conclusions

This work develops an unmanned system, uDSS, for juice sampling from tomatoes. The experiments have verified that uDSS is capable of performing all flight steps independently without the need for manual intervention. uDSS is able to arrive at the target field, identify target tomatoes, perform juice sampling, and return accurately and automatically. Within the task area, uDSS demonstrates good adaptability and stability. Meanwhile, the uDSS is user-friendly and affordable (~USD 1363). It is worth noting that this work is a proof of concept conducted within a limited area. Deployment over larger fields would require the anchor layout to be adapted and its positioning performance evaluated under the intended operating conditions. Relevant factors include anchor spacing and height, line-of-sight availability, signal interference and power supply. The present experiment does not establish an anchor count for hectare-scale deployment. Furthermore, uDSS performance may be impacted by strong winds or heavy rain, which could destabilize drone attitude or hinder visual recognition. Additionally, improvements remain possible in flight range, target recognition accuracy, and adaptability to complex environments. Future work will focus on scaling the system and strengthening design resilience against adverse weather conditions alongside adaptive control strategies to achieve efficient large-area navigation and precise directional sampling. The optimization of hardware and algorithms will further augment the application potential of uDSS. The 3D-printed microneedle is reusable after rinsing, and a fresh piece of synthetic paper is used for each sampling to reduce the risk of carryover between samples, which greatly reduces the cost per sample. In the future, we will continue to optimize and improve the design of the MN device to increase its adaptability for different vegetables and fruits. The uDSS can be expanded with additional sensors and devices for application in other scenarios, including pesticide and fertilizer sowing, as well as real-time monitoring of the growth status and yield of various crops, thereby enabling precision agricultural management. We believe that the uDSS will provide useful solutions for intelligent agriculture due to its scalability. Finally, it should be emphasized that this work is a proof of concept: the reported results were obtained with a limited number of tomatoes, the analysis of the sampling and puncture success rates is based on small groups of samples (Tables S2 and S3), and the experiments were carried out with a single tomato variety and under mild weather conditions. Further systematic evaluation with more independent fruits, additional varieties and a wider range of environmental conditions is needed to assess generalizability.

## Figures and Tables

**Table 1:** Comparison of the uDSS with representative UAV-based systems for agriculture and aerial interaction tasks.

System	Platform/Positioning	Payload and Interaction with the Object	Value Reported to the User
Primicerio et al. [18]	Six-rotor UAV, GNSS	Multispectral camera, non-contact sensing	Vineyard reflectance maps
Zhang et al. [20]	DJI S1000+, GNSS	Hyperspectral camera, non-contact sensing	Soybean plot yield prediction
Sheng et al. [37]	Multirotor UAV, manual supervision	Microneedle patch for drug delivery into animals	UAV-mediated first aid
This work (uDSS)	Quadrotor UAV, UWB + VIO + laser rangefinder	3D-printed microneedle with synthetic paper; physical penetration and capillary juice collection	Tomato juice sample, Brix value for maturity assessment

## Data Availability

Data are contained within the article or Supplementary Materials.

## Supplementary Materials

The following supporting information can be downloaded at: https://www.mdpi.com/article/10.3390/mi17101184/s1. The cost analysis of uDSS (Table S1), effect of tomato size on sampling and puncture success rates (within the same maturity range) (Table S2), effect of tomato ripeness on sampling and puncture success rates (within the same size range) (Table S3), structure of synthetic paper (Figure S1), architecture of the pressure sensor (Figure S2), AMS1117-3.3V circuit (Figure S3), CH340C circuit (Figure S4), schematic of the pressure sensor design design (Figure S5), testing of pressure sensors with diameters of 7.6, 10 and 18.3 mm (Figure S6), voltage comparator module (Figure S7) are included in the Supplementary Materials.
