# Supplementary material for: Rapid Sampling of Tomato Juice Using a 3D-Printed Microneedle Assisted by an Unmanned Aerial Vehicle
Source: Micromachines (Basel). 2026 Oct 13;17(10):1184. doi: 10.3390/mi17101184 (PMC13707942; doi:10.3390/mi17101184)
Supplement: Supplementary file 1 [file micromachines-17-01184-s001.zip › micromachines-4565494-supplementary.pdf]

Supplementary material

Rapid Sampling of Tomato Juice using a 3D-Printed Microneedle Assisted by an Unmanned Aerial Vehicle

Weixin Liu[1†], Tianyu Wang[1†], Jiahua Zhong[2], Qinghao He[2], Haonan Li[2,3,*], Weijin Guo[2,*]

[1]Department of Electronic Engineering, Shantou University, 243 Daxue Road, Shantou, 515063, Guangdong, China.

[2*]Department of Biomedical Engineering, Shantou University, 243 Daxue Road, Shantou, 515063, Guangdong, China.

[3*]School of Biomedical Engineering, Shenzhen Campus of Sun Yat-sen University, 66 Gongchang Road, Shenzhen, 518107, Guangdong, China.

†These authors contributed equally to this work.

*Corresponding author(s). E-mail(s): lihn28@mail2.sysu.edu.cn; guoweijin@stu.edu.cn;

Cost analysis of uDSS

Table S1 shows the cost analysis of the system. Detailed information of raw materials and electronic components needed per device are included in the table.

Table S1. Cost estimation of uDSS

Materials	Unit cost	Materials per Device	Cost per Device
**Onboard computer (Intel BXNUC10i7FNKN2)**	421.2 USD	1 unit	**421.2 USD**
**UAV Propeller**	1.4742 USD	4 units	**5.8968 USD**
**UAV motors (HKSRC 2205-2450KV)**	4.914 USD	4 units	**19.656 USD**
**Motor ESC (Formula 32 bit 45A)**	5.1948 USD	4 units	**20.7792 USD**
**LinkTrack S UWB**	56.16 USD	6 units	**336.96 USD**
**UAV flight control system (Holybro Pixhawk 6C)**	145.7352 USD	1 unit	**145.7352 USD**
**Pressure Sensor (PCB + RP-C10-ST)**	11.232 USD	1 unit	**11.232 USD**
**UAV Frame Q250**	11.934 USD	1 unit	**11.934 USD**
**Intel RealSense D435i Depth Camera**	351 USD	1 unit	**351 USD**
**USB Camera (DSJ-YC60Z)**	19.3725 USD	1 unit	**19.3725 USD**

**Distance measurement sensor (STP-23)**	17.9712 USD	1 unit	**17.9712 USD**
**OSTE**	0.127 USD/g	1.85g	**0.23495 USD**
**Silica Gel**	0.002808 USD/g	10 g	**0.02808 USD**
**PLA (for 3D printing)**	0.0096 USD/g	57.57 g	**0.5544 USD**
		Total：	**~1362.55433 USD**

Fabrication and dimensional tolerance of the 3D-printed microneedle

The microneedle was designed in Fusion 360 and printed with an FDM 3D printer (Creality K1) using PLA filament with a nozzle diameter of 0.4 mm, a layer height of 0.1 mm and an extrusion temperature of 210 °C. The needle was printed vertically to avoid support material on the shaft and to align the layer planes perpendicular to the penetration direction. The four functional dimensions of the design are the overall length of the needle (30 mm), the length of the needle section below the mounting flange (19.72 mm), the diameter of the tip (0.2 mm) and the width of the two synthetic paper slots (2.5 mm each); their rationale is given in Section 2.3.2, and the MN device and its components are shown in Fig. 5 of the main manuscript.

These dimensions are CAD design values. The printer settings describe the fabrication process and do not establish the measured dimensions or dimensional repeatability of the printed parts. Functional sampling performance is reported in Tables S2 and S3.

Because the tip diameter determines both the initial puncture force and the resistance to buckling, the tip region was additionally inspected optically after the sampling experiments: no deformation, fracture or detachment of the tip was observed on the needles recovered after a completed sampling mission, which indicates that the printed needle withstands both the puncture of the tomato skin and steps of the sampling procedure.

Structure of synthetic paper

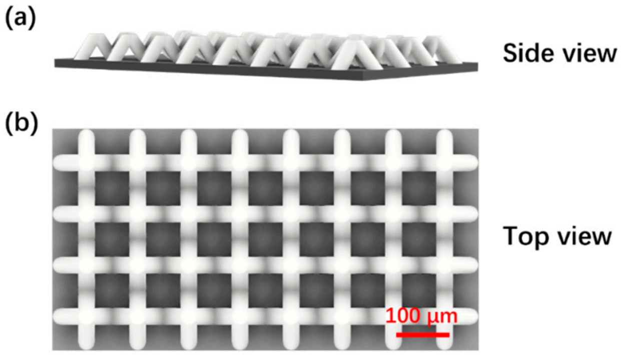

Figure S1. Structure diagram of synthetic paper. (a) Side view of synthetic paper. (b) Top view of synthetic paper.

Performance evaluation of the synthetic paper

The juice filling rate and the juice absorption capacity of the synthetic paper were measured gravimetrically. A piece of synthetic paper with an exposed area of 1.0 cm^2 was weighed on a balance with a resolution of 0.1 mg and then brought into contact with a reservoir of freshly extracted tomato juice at room temperature (25 °C). The mass of the sample was recorded at intervals of 1 s for 5 s, and the mass increment was converted into a volume of absorbed juice using the density of tomato juice (1.04 g/cm^3, measured with a pycnometer). The filling rate is defined as the ratio of the volume of juice absorbed within 5 s to the total pore volume of the sample; the total pore volume was obtained from the mass of the dry sample, the bulk volume of the sample and the density of the OSTE polymer (1.15 g/cm^3) measured by the buoyancy method. The absorption capacity is defined as the volume of juice absorbed per unit exposed area of the synthetic paper at saturation. Five independent samples were measured for each quantity, and the results are reported as mean ± standard deviation ($n = 5$).

The juice recovery rate was determined by centrifugation. A saturated sample was weighed, transferred into a centrifuge tube equipped with a filter insert, and centrifuged at 3000 rpm for 5 minutes; the mass of the juice collected at the bottom of the tube was then measured, and the recovery rate is defined as the ratio of the volume of juice recovered by centrifugation to the volume of juice absorbed by the sample before centrifugation. The measurement was repeated on the same piece of synthetic paper over five consecutive absorption-centrifugation cycles in order to assess the effect of repeated use on the recovery rate.

The open, interconnected micropillar structure supports capillary uptake of tomato juice. Absorption and recovery were evaluated using tomato juice, and no measurable decrease in recovery rate was observed over five consecutive absorption – centrifugation cycles under the tested conditions. These measurements characterize performance for the tested juice and do not establish general resistance to clogging across different pulp contents or viscosities. Fresh synthetic paper was used for each sampling to reduce carryover between samples. Moreover, the size of tomato seeds is larger than the pore size of synthetic paper, so synthetic paper actually acts as a filter during the process of absorbing tomato juice, and the tomato seeds cannot clog the pores of the synthetic paper.

Architecture of the pressure sensor

The pressure sensor module monitors the pressure of the MN device applied to the tomato by the drone in real time to facilitate the juice collection at a suitable pressure. The voltage comparator module provides another convenient pressure monitoring scheme for manually setting the pressure monitoring threshold and the over-pressure alarm lamp during the juice collection. The overall structure of the pressure sensor system is shown in Fig. S2.

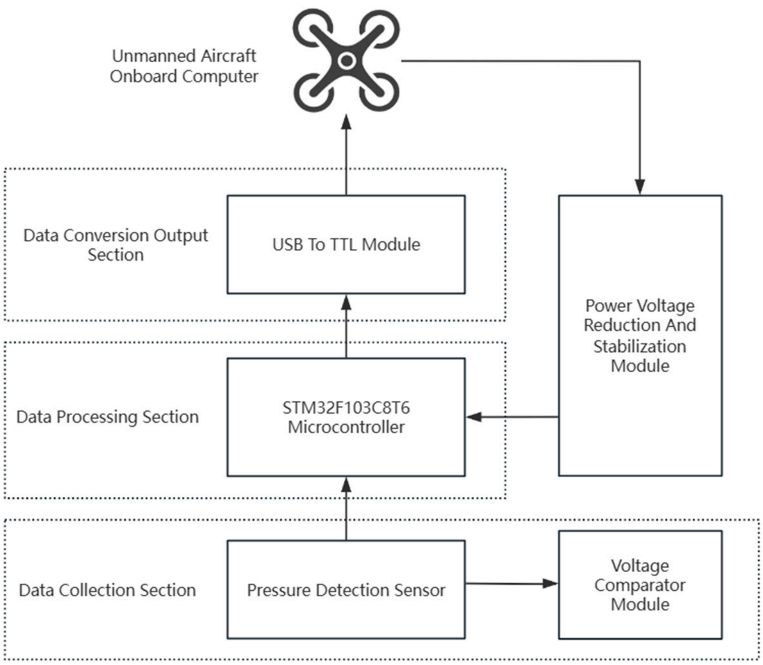

Figure S2. The overall structure of the pressure sensor system

Power supply and serial port signal conversion module of pressure sensor system

Considering the limited interfaces provided by the UAV airborne computer to the peripherals, the pressure sensor system is designed to integrate the two functions of serial data transmission and system power supply into a shared interface. Because the peripheral interface of the UAV airborne computer is a universal USB interface, the output voltage is 5.0 V, but the main control chip of the pressure sensor system works at 3.3 V, so the power supply step-down module needs to be added. The power supply step-down module adopts the AMS1117-3.3 V chip, which can reduce the DC power supply in the range of 4.75 V ~ 12 V to 3.3 V DC, and the maximum output current can reach 800 mA. The circuit of the chip in this system is shown in Fig. S3.

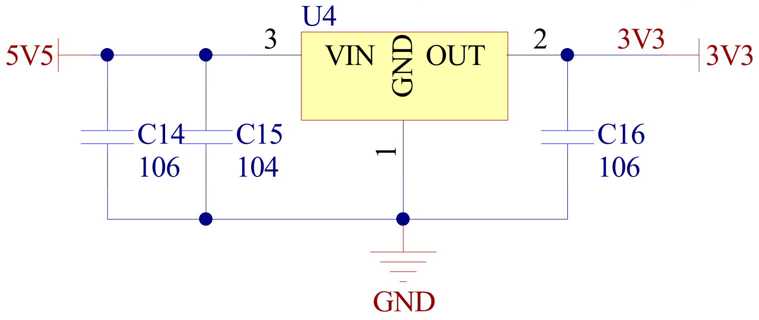

Figure S3. AMS1117-3.3V circuit

The peripheral interface provided by the UAV airborne computer is a universal USB interface. Only the USB type signal can be identified on the airborne computer,

but the signal generated by the main control chip of the pressure sensor is TTL type. In order to realize the communication between the airborne computer and the pressure sensor system, the serial port signal conversion module is integrated as the medium. The CH340 C chip is used in the serial signal conversion module, which can decode the data packets on the USB bus of the airborne computer to the serial data stream of the TTL level, and encode the serial data into USB data packets and send them to the host computer to realize the conversion between the serial port of the airborne computer and the pressure sensor system. The circuit of the chip in this system is shown in Fig. S4.

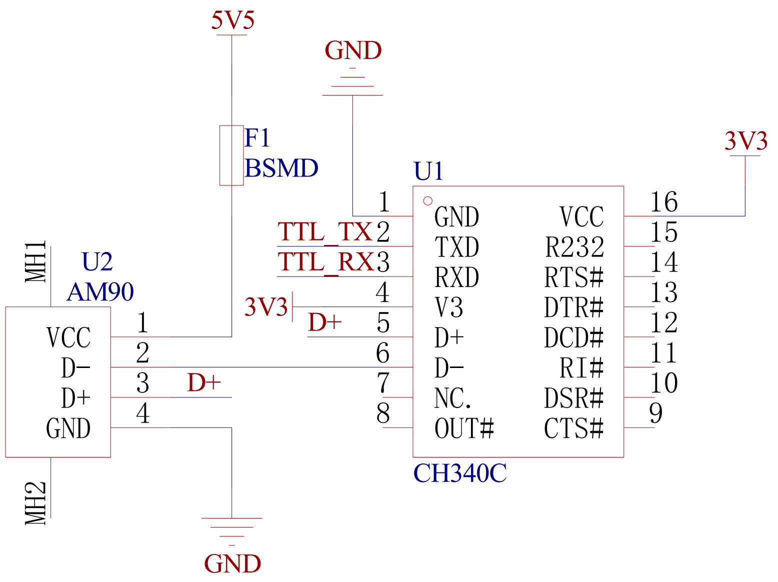

Figure S4. CH340C circuit

Design and testing of the pressure sensor

The pressure sensor module adopts the RP-C10-ST type film pressure sensor. This type of sensor is printed on a flexible polyester substrate with functional materials such as electrodes, force-sensitive layer, insulation glue, etc. The upper and lower layers of the substrate are packaged face-to-face to extract the electrode. The sensor is based on resistance change. The larger the force is, the smaller the resistance becomes. The structural diagram of the sensor is shown in Fig. S5.

The data acquisition function in the pressure sensor system mainly depends on the real-time detection of the analog-to-digital conversion of the voltage at both ends of the pressure detection sensor by the main controller of the system. The acquired voltage is correlated with the actual pressure to obtain real-time output pressure data. Here, we used pressure sensor patches with different diameter sizes (7.6 mm, 10 mm, 18.3 mm) combined with microneedle devices to test and compare the pressure values during penetrating tomatoes. We analyzed the pressure threshold and selected the pressure sensor patch with diameter as 10 mm for experiments. The pressure test diagram is shown in Fig. S6.

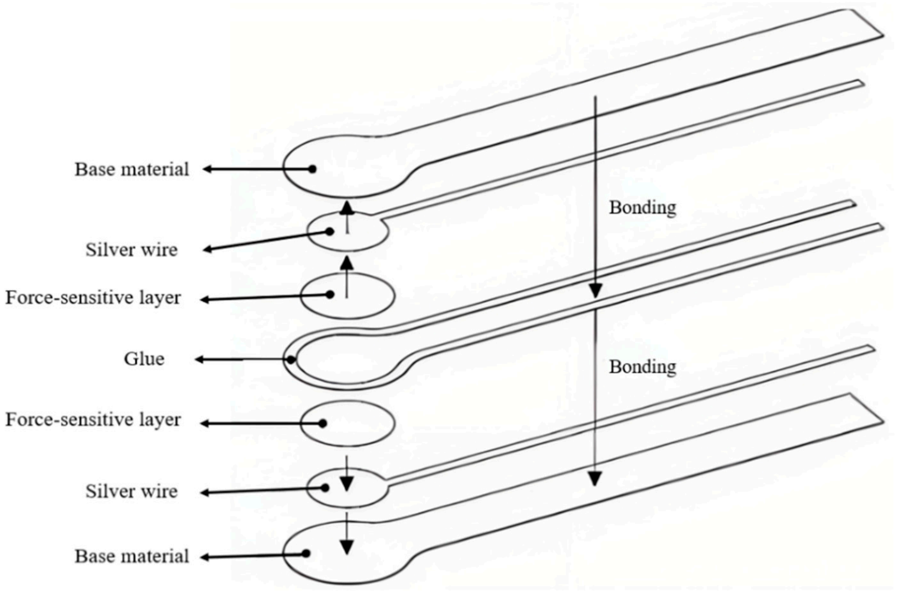

Figure S5. Schematic of the pressure sensor design

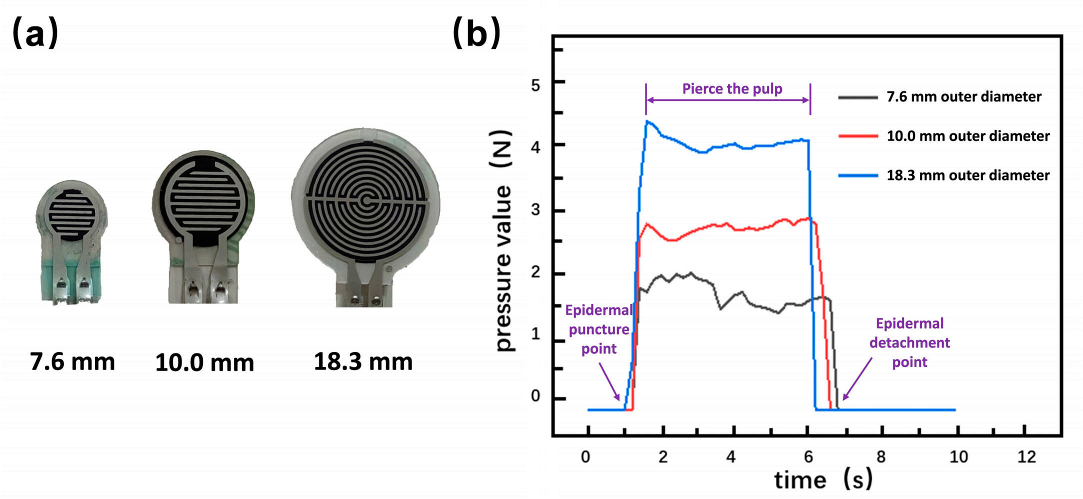

Figure S6. (a) Pressure sensors with diameters of 7.6, 10 and 18.3 mm. (b) Sensor responses during tomato puncture: black, red and blue curves correspond to the 7.6, 10 and 18.3 mm sensors, respectively. The 10 mm sensor was selected for the MN device.

Pressure sensor response and threshold setting

The 1.5 N threshold used in the sampling control sequence was selected based on preliminary independent penetration tests on the tomatoes examined in this work. The sensor signal was monitored during puncture, and the adjustable voltage comparator supplied the trigger for the juice-absorption step. The comparator circuit is shown in Fig. S7.

Fig. S6 compares the responses of sensors with diameters of 7.6, 10 and 18.3 mm. The 10 mm sensor was selected for the sampling experiments. These results describe the tested configuration; the threshold would require further evaluation before use with other tomato cultivars or ripeness ranges.

Voltage comparator module

We design a voltage comparator module for the pressure detection. The module uses the LM393 chip, which integrates two voltage comparators. In this system, one input end of the voltage comparator is connected to the voltage detection point of the pressure sensor, and the other input end is connected to the voltage detection point of an adjustable potentiometer. By detecting the potential change of the output end, it can be decided whether the pressure exceeds the threshold set by the potentiometer. The output end is connected to an LED circuit, and the internal real-time pressure detection can be visualized based on the LED light-off to facilitate debugging during the experiment. The circuit design of this part in the system is shown in Fig. S7.

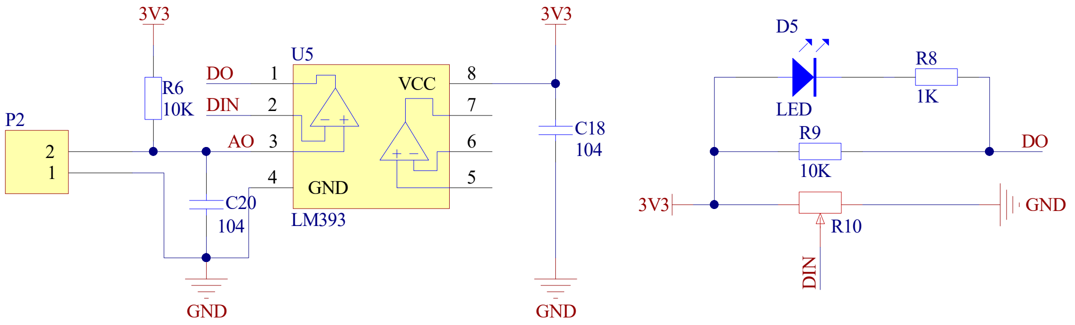

Figure S7. Circuit design of voltage comparator module

Fruit characteristics and sampling outcomes

We evaluated sampling and puncture outcomes for tomatoes of different sizes and ripeness levels. Tomatoes were categorized into three size groups: L (large; average diameter as 8.4 cm), M (medium; average diameter as 7.2 cm), and S (small; average diameter as 6.1 cm), and further classified by ripeness (sugar content, Brix value %). As shown in Tables S2 and S3, we tested tomatoes of different sizes at similar ripeness levels and tomatoes of similar sizes at different ripeness levels, evaluating their sampling success rate and puncture success rate respectively.

In these trials, medium tomatoes had the highest sampling success rate (10/15, 66.67%), whereas small tomatoes had the highest puncture success rate (7/8, 87.50%). It should be noted that the sampling success rate is determined by the first puncture and successful extraction of juice; the puncturing success rate is based on the microneedle contacting the fruit surface and successfully extracting juice on the synthetic paper. Table S2 includes five fruits per size group and 15 trials per group, whereas Table S3 includes three fruits per Brix group and six trials per group. The success rates in Tables S2 and S3 summarize trial outcomes. Because individual fruits were tested repeatedly, these results are reported descriptively and do not establish effects of fruit size or ripeness on sampling or puncture success.

Table S2. Effect of tomato size on sampling and puncture success rates (within the same maturity range)

**Fruit characteristics**	**L (average 8.4 cm) 7.5–8.9 cm**	**M (average 7.2 cm) 6.8–7.5 cm**	**S (average 6.1 cm) 5.7–6.8 cm**
**Maturity (Brix value, %)**	Average 3.6 (3.52–3.71)		
**Sample size and number of experiments**	5 samples for each size category, and 15 experiments for each fruit characteristic.		
**Sampling success rate**	53.33% (8/15)	66.67% (10/15)	46.67% (7/15)
**Puncture success rate**	72.73% (8/11)	83.33% (10/12)	87.50% (7/8)

Table S3. Effect of tomato ripeness on sampling and puncture success rates (within the same size range)

**Maturity (Brix value, %)**	**3.6**	**4.5**	**5.4**
**Fruit characteristics**	Average 7.2 cm (6.8–7.5 cm)		
**Sample size and number of experiments**	3 samples were selected from each maturity category, with 6 experiments conducted for each maturity category.		
**Sampling success rate**	66.67% (4/6)	66.67% (4/6)	83.33% (5/6)
**Puncture success rate**	80.00% (4/5)	80.00% (4/5)	100.00% (5/5)
